# Experimental Study on Thermal Decomposition Temperature and Thermal Expansion Coefficient of Typical Nonmetallic Materials in Aeroengine Components

**DOI:** 10.3390/ma18061250

**Published:** 2025-03-12

**Authors:** Bin Wu, Kai Wang, Tai Zeng, Wenguo Weng, Zuxi Xia, Zhengliang Su, Fei Xie

**Affiliations:** 1 The Second Research Institute of CAAC, Chengdu 610041, China; wubin@fccc.org.cn (B.W.);; 2 Key Laboratory of Aviation Fuel Airworthiness and Green Development of Civil Aviation, Chengdu 610041, China; 3 School of Safety Science, Institute of Public Safety Research, Tsinghua University, Beijing 100084, China; wgweng@tsinghua.edu.cn; 4 AECC Commercial Aircraft Engine Co., Ltd., Shanghai 200241, China

**Keywords:** thermal decomposition temperature, thermal expansion coefficient, aviation sealing material, aircraft fire safety

## Abstract

This paper aims to evaluate the thermal decomposition temperature and linear thermal expansion coefficient of typical non-metallic materials in aero-engine components. Thermogravimetric analysis and thermomechanical analysis were employed to systematically investigate the thermal and dimensional stability of these materials at varying heating rates, and their performance was validated through fireproof experiments. It was found that the high-strength graphite gasket exhibited the highest thermal decomposition temperature, while the polytetrafluoroethylene and fluorosilicone rubber showed excellent dimensional stability. Fluorine-based materials, such as fluorine rubber, showed higher thermal decomposition temperatures but relatively poor dimensional stability. This paper provides a scientific basis for the selection and design of sealing materials in aero-engines, contributing to the improvement of equipment safety and reliability.

## 1. Introduction

Aviation safety is one of the core concerns throughout the entire life cycle of civil aircraft, including design, manufacture, operation, and maintenance. Among these, fire is a hazardous event that can easily lead to serious aviation accidents, making preventive measures particularly crucial. Aircraft fires are often closely related to leaks in fuel and oil systems, and ensuring the integrity of these systems is the first line of defense against such leaks. Non-metallic seals play a vital role in key components of aircraft engines, such as auxiliary gearboxes (AGBs), slide tanks, and fuel metering units (FMUs). These seals must withstand the severe conditions of high temperatures and pressures in the event of a fire. The thermal stability of non-metallic materials is of paramount importance in maintaining the safe operation of fuel and oil systems. Seal failure is typically caused by material degradation at high temperatures. Therefore, it is particularly important to conduct a comprehensive and in-depth evaluation of the performance of non-metallic sealing materials under high-temperature conditions. Specifically, the thermal decomposition temperature and linear thermal expansion coefficient of the material are two key parameters that not only define the safe operating temperature range of the material but also directly reflect its dimensional stability in thermal environments. Through in-depth study and thorough understanding of these properties, the behavior of materials at high temperatures can be more accurately predicted, thereby providing a more robust foundation for aviation safety.

In terms of improving the thermal stability of materials, researchers have achieved remarkable results. Through extensive experimental research and theoretical analysis, scientists have not only revealed the complex behavior mechanisms of non-metallic materials at high temperatures but also proposed a variety of innovative solutions to optimize the properties of materials. These studies have deepened our understanding of how various high-performance materials behave in extreme environments and provided strong experimental and theoretical support for future materials science research. Wu et al. [1] dedicated their efforts to developing wear-resistant and high-temperature-resistant carbon fiber-reinforced silicone rubber/fluorine rubber (CF/MVQ/FKM) materials. By optimizing the type and content of coupling agents, they improved the mechanical and thermal properties of the materials, increasing the initial thermal decomposition temperature from 231 °C to 304 °C. Wu et al. [2] prepared a methyl vinyl silicone rubber/fluorine rubber (MVQ/FKM) mixture by mechanical mixing method and found that the material had the best mechanical properties at the MVQ: FKM ratio of 1:9. In addition, the thermal properties of the MVQ/FKM mixture are affected by its molecular structure, especially the difference between Si-O and C-C bond energies. Rahmani et al. [3] studied the thermal stability of fluoropolymers enhanced by carbon nanofibers through thermogravimetric analysis (TGA) and differential scanning calorimetry (DSC). Their results demonstrated that the introduction of carbon nanofibers could significantly improve the thermal stability of the material. Pires et al. [4] examined the thermal aging behavior of fluorosilicone rubber under high-temperature conditions, revealing that thermal aging would lead to changes in the mechanical properties of the materials, such as embrittlement and crosslinking. He et al. [5] employed multiple thermogravimetric analysis methods to study the thermal degradation characteristics of fluoroelastomers in detail, concluding that fluoroelastomers had good thermal stability under high-temperature environments. Alfannakh et al. [6] compared the thermal stability of ethylene-propylene diene monomer (EPDM) composites with and without lead powder using TGA, and found that lead power-containing composites displayed superior thermal stability at high temperatures. Nayak et al. [7] studied the thermal decomposition kinetics of polyimide materials reinforced with various carbon nanofillers, discovering that the introduction of nanomaterials significantly reduced the decomposition rate of polyimide and enhanced its thermal stability. Dong et al. [8] explored the thermal properties of graphene/natural rubber (GE/NR) nanocomposites, showing that graphene addition improved the thermal stability of NR. Zhu et al. [9] studied the thermal decomposition characteristics of cross-linked polycarbonate derived from carbon dioxide, propylene oxide, and dianhydride of pyrolytic acid, elucidating its thermal decomposition mechanism. Zhang et al. [10] analyzed the thermal decomposition behavior of brominated butyl rubber (BIIR) using varying heating rates, identifying key factors affecting its thermal decomposition. Erickson [11] studied the thermal decomposition mechanism of several polymers, emphasizing the critical role of polymer decomposition in fire dynamics. Prasad et al. [12] studied the thermal decomposition kinetics of fluoropolymers, using TGA and DSC, finding that varying heating rates significantly affected the polymer’s decomposition behavior. Ichihara et al. [13] employed continuous reaction thermogravimetric analysis to study the thermal decomposition behavior of fluororubbers, revealing a multi-stage reaction mechanism that provides deeper insights into improving the thermal stability of such materials. Bettinger et al. [14] further investigated the thermal decomposition characteristics of fluorinated single-walled carbon nanotubes under extreme temperatures. By conducting experiments under high vacuum conditions, they analyzed the gaseous decomposition products formed between 50 and 500 °C. The study revealed the primary decomposition product was carbonyl fluoride (COF2) below 300 °C, while CF dominated above 300 °C. Sokmen and Cakhusr [15] conducted numerical and experimental studies on the thermal expansion of air hoses made from different materials at high temperatures and its effect on pressure loss. They found that material selection had a great influence on the pressure loss. Korolev et al. [16] experimentally studied the thermal expansion behavior of thermosetting polymers and glass fiber reinforced plastics (GRP), uncovering their nonlinear thermal expansion characteristics under heating conditions. In the study of fire behavior and fire-retardant materials, Grange et al. [17] explored the fire behavior of carbon PEKK composites in aviation applications. Their experiments showed that the material maintained structural integrity under high heat flux and limit fire spread. Krawiec et al. [18] evaluated the safety of drive and transport belt materials from the perspective of thermal decomposition and toxicity, especially the risk of toxicity emissions under high-temperature conditions. Qi et al. [19] studied the tribological properties of PTFE/fluorosilicone rubber/silicone rubber composites at high temperatures, finding that high temperatures significantly improved the friction stability and wear resistance of the materials, which is of great value for the development of high-performance sealing applications. Pinedo et al. [20] conducted a study on the thermal analysis and tribological behavior of thermoplastic polyurethane and nitrile butadiene rubber in sealing applications, revealing the significant effect of temperature on these materials’ properties.

The fire protection performance of the fuel system is a key aspect of aircraft fire safety design. Various aviation regulations and standards have set strict requirements for fire testing methods to verify the fireproof of fuel system components in extreme high-temperature environments. FAA 14 CFR Part 25 [21] and EASA CS-25 [22] explicitly state that fuel tanks and their related systems must be capable of withstanding ignition risks caused by lightning, fault currents, and single-point failures. Furthermore, the design of fuel tanks must prevent fuel leakage into areas that could lead to fires and ensure that any potential leaks do not come into contact with high-temperature surfaces or ignition sources. Regarding fuel delivery systems, SAE ARP 1179 [23] specifies fire protection requirements for fuel delivery pipelines, fittings, and seals, ensuring that the fuel system maintains basic functionality under high-temperature exposure and prevents fuel leaks caused by material degradation or thermal stress. ISO 2685 [24] provides specific testing methods for the fire performance of aviation equipment, requiring that fuel system components undergo fireproof and fire-resistant tests using standard flame burners. For fireproof tests, the sample must be exposed to a high-temperature flame environment of 1100 °C (±80 °C) for 15 min, while the fire-resistant test lasts for 5 min. These tests assess the integrity and leak protection capabilities of different levels of fuel system components under high-temperature flame exposure. RTCA DO-160 [25] establishes environmental adaptability requirements for fire and flammability testing of airborne equipment, emphasizing that the fuel system should maintain structural integrity under both normal and fault conditions, and prevent fuel from entering areas that could lead to fires. Overall, these regulations and standards together form a technical framework for fuel system fire protection, ensuring that aircraft are effectively safeguarded against safety risks posed by fuel leaks during design, manufacturing, and operation, providing a systematic technical guarantee for improving the overall fire protection performance of aviation fuel systems.

The seals of aircraft engine fuel and lubricating oil systems are primarily made from non-metallic materials. Seal failure can lead to liquid leakage inside the pipelines, which can result in severe consequences. High temperature and thermal aging are among the primary causes of failure in non-metallic material seals. In high-temperature environments, the polymer chains in non-metallic materials break due to thermal degradation, while low molecular weight additives in the material may volatilize or migrate. These changes cause the material’s hardness to increase, strength to decrease, contact stress to reduce, and dimensional stability to worsen, significantly impairing sealing performance. Therefore, measuring the thermal properties of non-metallic materials is crucial for evaluating their sealing performance in high-temperature environments. The aim of this paper is to experimentally determine the thermal decomposition temperature and linear thermal expansion coefficient of several typical non-metallic materials—fluoro rubber (FKM), fluorocarbon rubber (FKM), fluoro ether rubber (FFKM), fluoro silicone rubber (FVMQ), nitrile butadiene rubber (NBR), polytetrafluoroethylene (PTFE), and high strength graphite gaskets (HSGs)—used for rubber seals engine parts. Through these data, the performance of these aviation materials in practical applications can be better evaluated, thus providing a scientific basis and key data for flight safety and equipment maintenance. It is worth noting that the dimensional stability of seals at high temperatures is not directly related to their sealing properties, but it can provide basic data for the analysis of combustible non-metallic fire surface in this paper. This is because dimensional stability is a basic physical property, while the sealing property is a functional property, influenced not only by the nature of the sealing material itself but also by the working environment, seal structure, assembly process and other factors.

## 2. Experimental Process

### 2.1. Object of Study

In the operating environment of aircraft engines, extreme conditions such as high temperature, high pressure, chemical corrosion, and mechanical stress necessitate the use of high-performance sealing materials. This paper focuses on eight types of non-metallic materials: FKM, FFKM, FVMQ, NBR, PTFE, and HSGs, with their key properties summarized in Table 1, Table 2 and Table 3. FKM is widely used in sealing components for fuel, lubrication, and hydraulic systems due to its excellent high-temperature and chemical resistance. FFKM performs exceptionally well in extreme high-temperature and highly corrosive environments, making it ideal for sealing components in combustion chambers and turbine sections. FVMQ combines both high and low-temperature resistance, making it suitable for low-temperature sealing and electrical insulation applications. NBR, known for its good oil resistance and mechanical properties, is primarily used in sealing components and fuel system parts under low-pressure and moderate-temperature conditions. Additionally, PTFE, with its extremely low friction coefficient, excellent chemical inertness, and non-stick properties, is commonly used for low-friction seals, coatings, and electrical insulation. Graphite, valued for its high-temperature resistance, self-lubricating properties, and conductivity/thermal conductivity, plays a critical role in high-temperature seals, bearings, and thermal management materials.

### 2.2. Thermal Decomposition Temperature

#### 2.2.1. Basic Principle of Experiment

The basic principle of the TGA for measuring thermal decomposition temperature is to study the thermal stability and decomposition characteristics of a sample by monitoring the change in its mass during heating in real time. In the experiment, the sample to be tested is placed on the sample tray of the instrument and heated at a constant heating rate. As the temperature increases, the sample undergoes physical or chemical changes such as volatilization, decomposition, or melting, which result in a gradual decrease in mass. The instrument continuously monitors the change in mass with a precise electronic balance and records the relationship between mass and temperature, forming a thermogravimetric curve. By analyzing this curve, especially the temperature points where mass sharply decreases, the thermal decomposition temperature of the sample can be accurately determined. This method not only reveals the material’s pyrolysis process but also provides important information about its thermal stability, decomposition behavior, and performance in high-temperature environments.

#### 2.2.2. Experimental Steps

At present, the standard for analyzing the critical temperature of thermal stability of polymers by TG curves are not uniform. The main research parameters include the initial weight loss temperature, the extrapolated initial weight loss temperature (the intersection of the maximum slope point tangent and the baseline), the end weight loss temperature, the maximum weight loss rate temperature, and the predetermined percentage weight loss temperature. In this paper, the thermal stability of non-metallic materials involved in typical engine components was analyzed using the pyrolysis characteristics of percentage weight loss temperature (1%, 5%, 10% and 50%), *T*_e_ (extrapolated initial weight loss temperature), *T*_m_ (maximum weight loss rate temperature), and other parameters.

In the thermal decomposition temperature experiment, two varying heating rates were set for each non-metallic material: 10 °C/min and 20 °C/min. The gas flow used in the experiment was air, with a flow rate of 50 mL/min, and the temperature range covered from 30 °C to 800 °C. All experiments were conducted using the TGA (Mettler TGA 2 Thermogravimetric Analyzer, Zurich, Switzerland) [33]. The main performance parameters of the instrument are listed in Table 4.

In the preparation before the experiment, it is necessary to record the ambient temperature and pressure and conduct a comprehensive inspection of the equipment to complete the mass and temperature calibration of the equipment. During the sample preparation process, the non-metallic material to be measured is wiped by anhydrous ethanol to remove particles and then placed in a dryer at 23 °C ± 5 °C for more than 24 h. During sampling, the sample is weighed and placed in the sample dish. After adjusting the thermobalance to zero, the sample dish is placed on the thermobalance. The gas flow rate is selected, the air flow is initiated, and the initial mass is recorded. The temperature program is set, including the initial temperature, the termination temperature and the heating rate. After the experimental starts, the temperature program is run, and the thermogravimetric curve is recorded. Finally, data processing and result analysis were carried out, the thermogravimetric data are displayed as a curve showing the relationship between mass change (or mass fraction change). The temperature values corresponding to 5%, 10%, 50% weight loss, as well as the temperature at the maximum weight loss rate, are recorded.

### 2.3. Linear Thermal Expansion Coefficient

#### 2.3.1. Basic Principle of Experiment

The dimensional stability of a material refers to the performance of a material that does not change its external dimension under mechanical force, heat, or other external influences. In general, heating a material causes it to expand thermally. The coefficient of thermal expansion refers to the characteristic that that describes how the geometric properties of a substance change with temperature due to thermal expansion and contraction. It is used to characterize the thermal expansion behavior of the material. Generally, the smaller the linear coefficient of thermal expansion, the better the dimensional stability of the material. For example, the linear coefficient of thermal expansion of polymer materials is 100–300 × 10^−6^ °C^−1^, that for metal ranges from 3 to 20 × 10^−6^ °C^−1^, and that for ceramic materials is around 3 to 5 × 10^−6^ °C^−1^.

#### 2.3.2. Experimental Steps

In this paper, the temperature deformation curve, the average linear thermal expansion coefficient, and the differential linear thermal expansion coefficient are used to analyze the geometric characteristics of several non-metallic materials under the condition of temperature increase. TA Q400EM (TA Instruments, New Castle, DE, USA) thermomechanical analyzer (TMA) [34]. The main performance parameters of the instrument are listed in Table 5. For each material, two heating rates of 10 °C/min and 20 °C/min were used, with air as the gas flow, a flow rate of 50 mL/min, a temperature range of 30 °C to 400 °C, and a preload force of 0.01 N.

At the stage of sample preparation, the rubber material should be processed into a rectangular standard sample with a length of 10 mm and a width of 10 mm. During the instrument calibration process, the general calibration steps should be performed, including force calibration, probe calibration, temperature calibration and furnace constant calibration. To ensure the consistency of the sample state, the following specific operation is required: heat the sample from 50 °C below its glass transition temperature to 50 °C above its glass transition temperature, maintain this temperature for 5 min, and then cool it to 30 °C at the actual experimental cooling rate. Before entering the experimental phase, check the state of the equipment and record the current ambient temperature and pressure. Then, clean the sample surface, probe, and sample holder. Place the sample on the sample holder, and apply 0.01 N load to ensure that the experimental probe is close to but not in contact with the sample surface. During the experiment, the sample is placed in a constant flow of air with a flow rate of 50 mL/min, and the temperature is increased at a constant rate. Record the TMA curve, which shows how the sample dimensions change as the temperature increases. Finally, process the data and analyze the results.

### 2.4. Material Fireproof Testing

#### 2.4.1. Experimental Equipment

**Figure 1:**
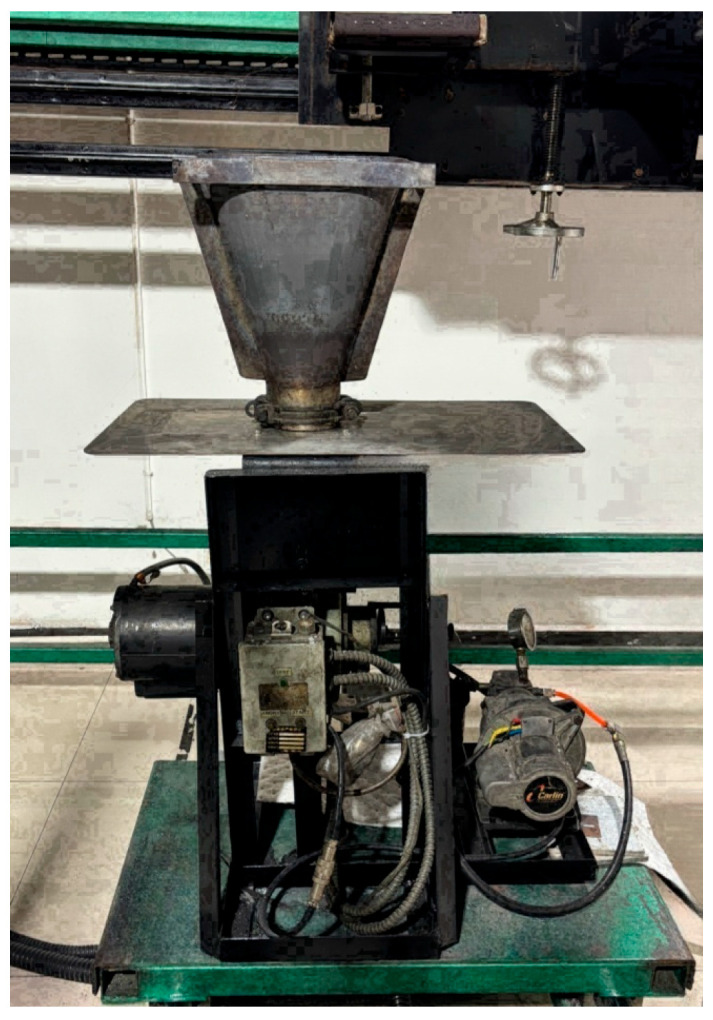
Photograph of the Carlin 201 CRD burner.

The Carlin 201 CRD burner is a high-precision combustion device specifically designed for aircraft fire safety testing. It is capable of simulating fire scenarios in aviation environments and is used to assess the fireproof of materials and components. The burner features precise control functions that allow adjustment of flame temperature, heat flux, and combustion time, meeting testing standards such as FAA and ISO (e.g., FAR 25.853 and ISO 2685). It is widely used in the fire performance testing of aircraft interior materials (such as seats, carpets, and partitions), engine components (such as engine compartments and fuel systems), and composite materials. The burner provides a realistic fire simulation environment to ensure the reliability and repeatability of test results. In this study, the fire source equipment used is the Carlin 201 CRD burner, as shown in Figure 1.

#### 2.4.2. Flame Calibration

**Figure 2:**
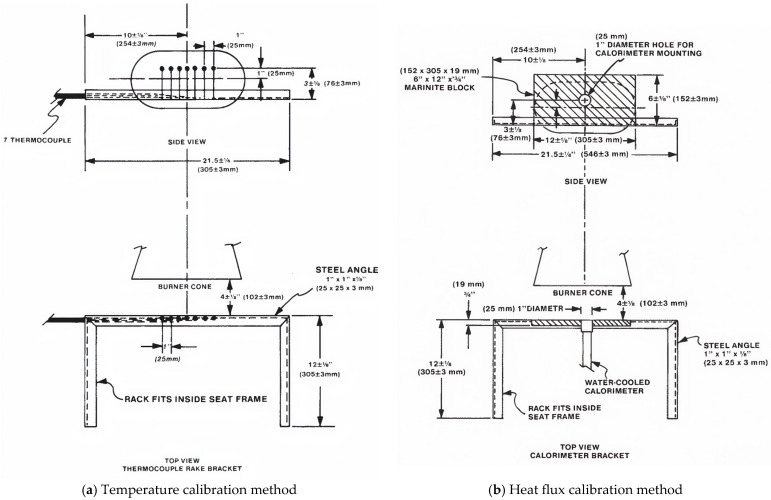
Schematic diagram of the calibration method for burner flame temperature and heat flux compliant with FAA standards [21].

The method and steps for conducting aircraft fire safety experiments are as follows: First, fix the thermocouple comb onto the test bench and place the burner in the preheating position, ensuring that the distance between the burner outlet and the fire-exposed surface of the test specimen is 102 mm ± 2 mm, as shown in Figure 2; after igniting the burner, preheat for 5 min to stabilize the flame, then move the thermocouple comb to the test position, collect temperature data from the 7 thermocouples (sampling frequency of 1 s, for a duration of 30 s), calculate the average value, and confirm that the flame temperature reaches 1090 °C ± 80 °C; next, move the thermocouple comb back to the preheating position and place the heat flux meter at the test position, confirming that the heat flux value is at least 10.6 W/cm^2^, and adjust the burner if the temperature or heat flux values do not meet the required standards; finally, once the flame calibration is complete, install the test specimen onto the test bench, ignite the burner, and begin the experiment [21].

**Figure 3:**
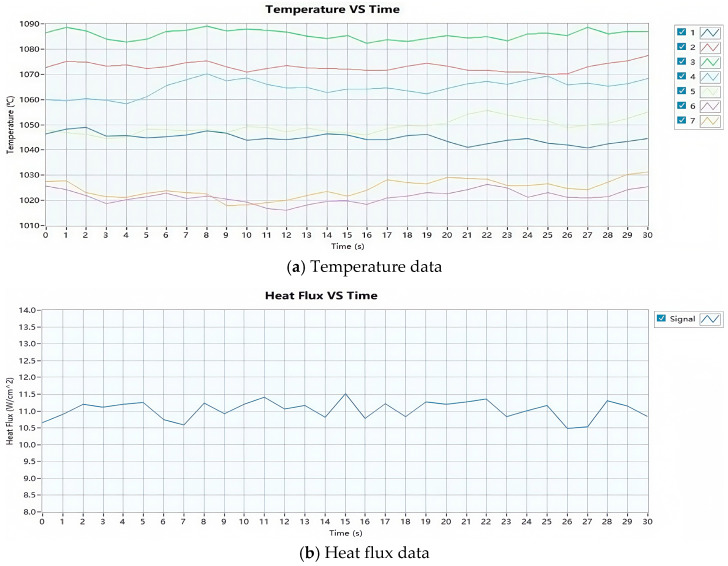
Temperature and heat flux calibration curves for the Carlin 201 CRD burner flame.

The flame calibration results are shown in Table 6 and Figure 3. It can be observed that the temperatures of the seven thermocouples are all within the range of 1000 °C to 1100 °C, with an average temperature of 1052 °C. The average heat flux density is 11 W/cm^2^, which meets the standard requirements for experimental flame temperature and heat flux.

## 3. Uncertainty Analysis

### 3.1. Uncertainty Analysis of Mass Loss and Pyrolysis Temperature

#### 3.1.1. TG

The uncertainty analysis in this study was conducted in accordance with ISO/IEC Guide 98-1:2024 [35]. The mathematical expression for the TG (Thermogravimetric) curve can be represented as [36]:

(1) $$\Delta m(T)=\left(\frac{m\left(T\right)-m_{0}}{m_{0}}\right)\times 100\%$$

where

*m*(*T*): the mass of the sample at temperature *T*;
*m*_0_: initial mass.

The principal sources of uncertainty influencing the percentage change in mass as a function of temperature include: uncertainties in mass measurement, such as the weighing accuracy, precision, repeatability, and reproducibility of the blank curve of the balance; uncertainties in temperature measurement, such as the accuracy and precision of the temperature sensor; uncertainties in the initial mass of the sample, such as measurement errors of the initial mass; and uncertainties in experimental conditions, such as variations in heating rates and atmosphere control, among other experimental parameters.

The uncertainty in mass measurement can be expressed as:

(2) $$u\left[m(T)\right]=\sqrt{{\left[a\times m(T)\right]}^{2}+{\left[b\times m(T)\right]}^{2}+c^{2}+d^{2}}$$

where

*a*: Weighing Accuracy;
*b*: Weighing Precision;
*c*: Repeatability;
*d*: Blank Curve Reproducibility.

The uncertainty of the initial mass measurement can be represented as:

(3) $$u\left[m_{0}\right]=\sqrt{{\left[a\times m_{0}\right]}^{2}+{\left[b\times m_{0}\right]}^{2}+c^{2}+d^{2}}$$

The uncertainty in temperature measurement can be expressed as:

(4) $$u(T)=\sqrt{e^{2}+f^{2}}$$

where

*e*: Temperature Accuracy;
*f*: Temperature Precision.

The uncertainty in the percentage of mass can be calculated using the following formula:

(5) $$u\left[\Delta m\left(T\right)\right]=\sqrt{{\left(\frac{\partial \left(\Delta m\left(T\right)\right)}{\partial m\left(T\right)}\cdot u\left[m\left(T\right)\right]\right)}^{2}+{\left(\frac{\partial \left(\Delta m\left(T\right)\right)}{\partial m_{0}}\cdot u\left(m_{0}\right)\right)}^{2}}$$

where

(6) $$\frac{\partial \left(\Delta m\left(T\right)\right)}{\partial m\left(T\right)}=\frac{1}{m_{0}}$$

(7) $$\frac{\partial \left(\Delta m\left(T\right)\right)}{\partial m_{0}}=-\frac{m\left(T\right)}{{m_{0}}^{2}}$$

The uncertainty of the mass percentage is given by:

(8) $$u\left[\Delta m\left(T\right)\right]=\sqrt{{\left(\frac{1}{m_{0}}\cdot u\left[m\left(T\right)\right]\right)}^{2}+{\left(\frac{m\left(T\right)}{{m_{0}}^{2}}\cdot u\left(m_{0}\right)\right)}^{2}}$$

Upon substituting the device parameters, the uncertainty in the mass percentage as a function of temperature is found to be 0.00753%.

#### 3.1.2. DTG

The mathematical expression for the DTG (Derivative Thermogravimetric) curve can be formulated as:

(9) $$\mathrm{DTG}(T)=\frac{d\left(\Delta m\left(T\right)\right)}{dT}$$

Since the DTG curve is the derivative of the TGA curve, the uncertainty of the DTG can be calculated through the uncertainty propagation formula for derivative operations. According to the uncertainty propagation formula, the uncertainty of the DTG curve can be expressed as:

(10) $$u\left(\mathrm{DTG}(T)\right)=\sqrt{{\left(\frac{\partial \left(\mathrm{DTG}\left(T\right)\right)}{\partial \left(\%\Delta m\left(T\right)\right)}\cdot u\left(\Delta m\left(T\right)\right)\right)}^{2}+{\left(\frac{\partial \left(\mathrm{DTG}\left(T\right)\right)}{\partial T}\cdot u\left(T\right)\right)}^{2}}$$

Upon incorporating the device parameters, the uncertainty associated with the mass percentage as a function of temperature is determined to be 0.00677% °C^−1^.

### 3.2. Uncertainty Analysis of the Coefficient of Linear Thermal Expansion

#### 3.2.1. Mean Coefficient of Linear Thermal Expansion

According to the temperature-deformation curve of the material, the linear thermal expansion coefficient can be calculated. Depending on the data processing method, the linear thermal expansion coefficient can be divided into the average linear thermal expansion coefficient and the differential linear thermal expansion coefficient.

Average linear thermal expansion coefficient is given by [37]:

(11) $$\alpha =\frac{\Delta L}{\Delta T}\times \frac{1}{L_{0}}$$

where

Δ*L*: the change of sample length in the measurement direction between temperature *T*_1_ and *T*_2_;

Δ*T*: the change in temperature, equal to *T*_2_ − *T*_1_;

*L*_0_: the initial length of the specimen in the measured direction at room temperature.

The uncertainty in the coefficient of linear thermal expansion can be determined through the application of the uncertainty propagation formula:

(12) $$u\left(\alpha \right)=\sqrt{{\left(\frac{\partial \alpha }{\partial \left(\Delta L\right)}\cdot u\left(\Delta L\right)\right)}^{2}+{\left(\frac{\partial \alpha }{\partial L_{0}}\right)}^{2}+{\left(\frac{\partial \alpha }{\partial \left(\Delta T\right)}\cdot u\left(\Delta T\right)\right)}^{2}}$$

where

(13) $$\frac{\partial \alpha }{\partial \left(\Delta L\right)}=\frac{1}{L_{0}\cdot \Delta T}$$

(14) $$\frac{\partial \alpha }{\partial L_{0}}=-\frac{\Delta L}{{L_{0}}^{2}\cdot \Delta T}$$

(15) $$\frac{\partial \alpha }{\partial \left(\Delta T\right)}=-\frac{\Delta L}{L_{0}\cdot \Delta T^{2}}$$

The uncertainty of the mean coefficient of linear thermal expansion is:

(16) $$u\left(\alpha \right)=\sqrt{{\left(\frac{u\left(\Delta L\right)}{L_{0}\cdot \Delta T}\right)}^{2}+{\left(\frac{\Delta L\cdot u_{0}\left(L_{0}\right)}{{L_{0}}^{2}\cdot \Delta T}\right)}^{2}+{\left(\frac{\Delta L\cdot u\left(\Delta T\right)}{L_{0}\cdot \Delta T^{2}}\right)}^{2}}$$

Upon substituting the equipment parameters, the uncertainty associated with the mean coefficient of linear thermal expansion is found to be 1.256 × 10^−7^ °C^−1^.

#### 3.2.2. Differential Coefficient of Linear Thermal Expansion

Differential linear coefficient of thermal expansion:

(17) $$\alpha_{\mathrm{diff}}=\frac{dL}{dT}\times \frac{1}{L_{0}}$$

where

d*L*: under constant pressure, the change of length during the experimental interval;

d*T*: under constant pressure, the change of temperature during the experimental interval;

*L*_0_: the initial length of the sample in the measurement direction at room temperature;

The uncertainty of the differential coefficient of linear thermal expansion can be calculated using the following formula:

(18) $$u\left(\alpha_{\mathrm{diff}}\right)=\sqrt{{\left(\frac{\partial \left(\alpha_{\mathrm{diff}}\right)}{\partial \alpha }\cdot u\left(\alpha \right)\right)}^{2}+{\left(\frac{\partial \left(\alpha_{\mathrm{diff}}\right)}{\partial T}\cdot u\left(T\right)\right)}^{2}}$$

Upon substituting the device parameters, the uncertainty in the differential coefficient of linear thermal expansion is calculated to be 1.382 × 10^−8^ °C^−1^.

## 4. Experimental Results

### 4.1. F117

**Figure 4:**
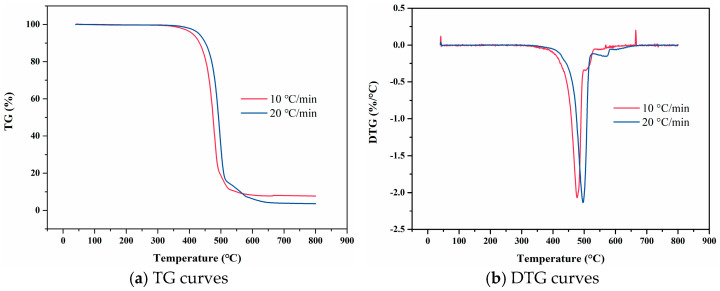
TG and DTG curves of F117 at varying heating rates.

As shown in Figure 4, TG curves at varying heating rates have similar shapes, and DTG curves all display a single main peak, indicating that the thermal decomposition of F117 involves only one primary reaction. When the heating rate is 10 °C/min, the TG curve remains nearly linear before 346 °C. However, beyond 410 °C, F117 undergoes a more pronounced thermal decomposition reaction. As the temperature increases, the thermal decomposition weight loss rate gradually increases, peaking at 477 °C, after which it gradually decreases until the thermal decomposition reaction ends. Similarly, at a heating rate of 20 °C/min, the TG curve is nearly linear before 371 °C. Beyond 430 °C, F117 exhibits a significant thermal decomposition reaction. The weight loss rate gradually increased with temperature, reaching the peak at 498 °C, and then gradually declines until the decomposition reaction ends. The heating rate is an important factor affecting the pyrolysis process. For the same polymer chain segment, an increase in heating rate causes the relaxation time of molecular chain movement to lag behind the experimental observation time. This phenomenon manifests as a shift in the weight loss temperature toward high temperature. Consequently, with the increase of heating rate, both the TG and DTG curve shows an overall shift toward higher temperature regions. In addition, the increase of heating rate has minimal impact on the volatilization yield of F117 during thermal decomposition, indicating that the main factor affecting the pyrolysis yield is temperature rather than heating rate.

**Figure 5:**
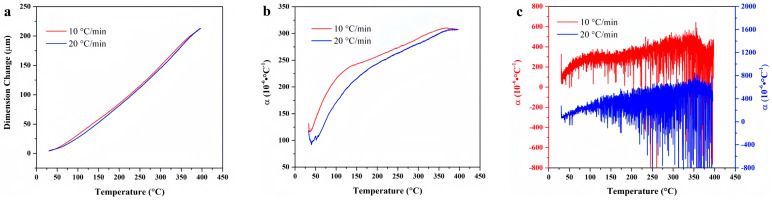
Temperature-deformation curves, temperature-average linear thermal expansion coefficient curves, and temperature-differential linear thermal expansion coefficient curves of F117 under varying heating rates. (**a**) Temperature-deformation curves; (**b**) Temperature-average linear thermal expansion coefficient curves; (**c**) Temperature-differential linear thermal expansion coefficient curves.

Figure 5 illustrates the temperature-deformation curves and temperature-linear thermal expansion coefficient curves of F117 at varying heating rates. When the heating rate is 10 °C/min, the linear thermal expansion coefficient is higher than that when the heating rate is 20 °C/min. This discrepancy can be attributed to the fact that the thermal expansion behavior of rubber is related to the diffusion movement of polymer chain segments. When the heating rate increases, the relaxation time of the molecular chain segment movement lags behind the experimental observation time, and the experimental value will be smaller. The average linear thermal expansion coefficient of F117 gradually increases with temperature and tends to be gentle at 370 °C, while the differential linear thermal expansion coefficient begins to decline near 370 °C. This indicates that the material is no longer expanding due to structural changes caused by thermal decomposition at high temperature.

### 4.2. FKM

**Figure 6:**
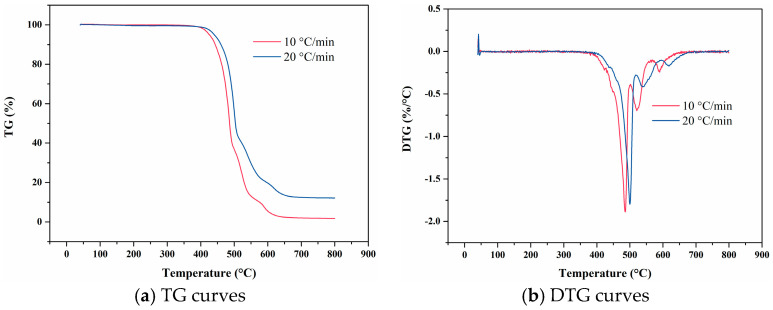
TG and DTG curves of FKM under varying heating rates.

Figure 6 presents the TG and DTG curves of FKM. The TG curves exhibit similar profiles across varying heating rates, indicating that the thermal decomposition behavior of FKM is largely consistent. The DTG curves each display three distinct peaks, indicating that FKM undergoes three thermal decomposition reactions. As the heating rate increases, the experimental decomposition temperature corresponding to the DTG peak shifted to higher temperatures, and the thermal decomposition temperature associated with the same weight loss rate also increased. For instance, when the heating rate increases from 10 °C/min to 20 °C/min, the temperature of the first maximum decomposition peak (*T*_m1_) increases from 485 °C to 500 °C. This observation indicates that the thermal decomposition temperature of FKM is closely related to the heating rate.

**Figure 7:**
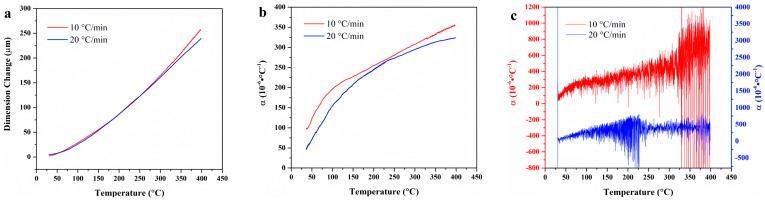
Temperature-deformation curves, temperature-average linear thermal expansion coefficient curves and temperature-differential linear thermal expansion coefficient curves of FKM under varying heating rates. (**a**) Temperature-deformation curves; (**b**) Temperature-average linear coefficient of thermal expansion curves; (**c**) Temperature-differential linear thermal expansion coefficient curves.

Figure 7 presents the temperature-deformation curve and temperature-linear thermal expansion coefficient curve of FKM. The linear thermal expansion coefficient at the heating rate of 10 °C/min is higher than that at the heating rate of 20 °C/min. Moreover, the thermal hysteresis resulting from increased heating rate is evident. Notably, the average and differential linear thermal expansion curves at the heating rate of 10 °C/min exhibit irregularities within this temperature segment. This irregularity suggests that the material has undergone significant thermal decomposition after prolonged heating, leading to an unstable thermal expansion coefficient that fluctuates in a discernible pattern.

### 4.3. FM-2D

**Figure 8:**
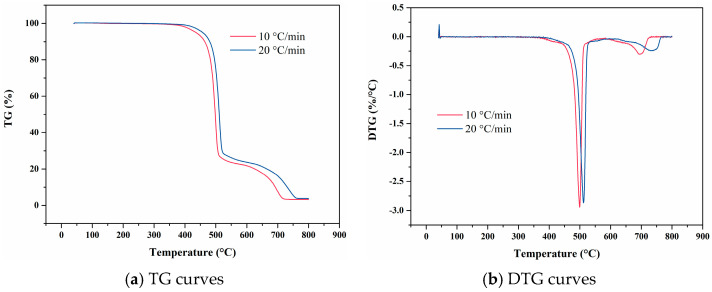
TG and DTG curves of FM-2D at varying heating rates.

Figure 8 presents the TG and DTG curves of FM-2D. The presence of two peaks in the DTG curve indicates that the decomposition process of FM-2D occurs in two distinct stages. The thermal decomposition behavior of FM-2D is consistent across varying heating rates. As the heating rate increases, the thermal decomposition temperature shifts to a higher temperature, and the peak temperature (*T*_m_) also increases. At a heating rate of 10 °C/min, the TG curve remains nearly linear up to 376 °C. From 430 °C to 500 °C, FM-2D undergoes a more pronounced thermal decomposition reaction, which corresponds to the primary weight loss stage. Additionally, a secondary weight loss stage occurs near 700 °C as the temperature continues to rise.

**Figure 9:**
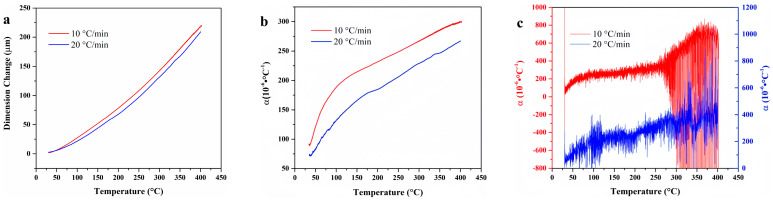
Temperature-deformation curves, temperature-average linear thermal expansion coefficient curves and temperature-differential linear thermal expansion coefficient curves of FM-2D under varying heating rates. (**a**) Temperature-deformation curves; (**b**) Temperature-average linear coefficient of thermal expansion curves; (**c**) Temperature-differential linear thermal expansion coefficient curves.

Figure 9 presents the temperature-deformation curve and temperature-linear thermal expansion coefficient curve of FM-2D. The linear thermal expansion coefficient at a heating rate of 10 °C/min is higher than that at a heating rate of 20 °C/min. This difference is attributed to the pronounced thermal hysteresis observed with an increased heating rate. As a result, the discrepancy between the average linear thermal expansion coefficients at varying heating rates can reach 50 × 10^−6^ °C^−1^. For the high-temperature range above 350 °C, the average linear thermal expansion curve of FM-2D at the heating rate of 10 °C/min is rough, which is associated with the decomposition of the material and the generation of gas after prolonged heat exposure.

### 4.4. FVMQ

**Figure 10:**
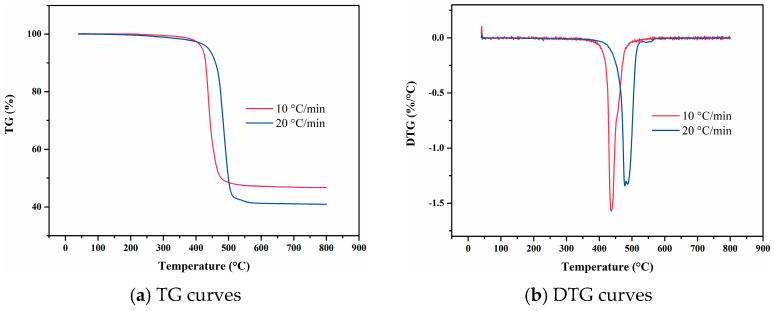
TG and DTG curves of FVMQ at varying heating rates.

As illustrated in Figure 10, the TG curves obtained at varying heating rates exhibit similar profiles, while the corresponding DTG curves each exhibit a single peak. This indicates that FVMQ undergoes a singular thermal decomposition mechanism, specifically the scission of the main chain. At a heating rate of 10 °C/min, the TG curve remains nearly linear up to 355 °C, indicating minimal weight loss and thermal stability within this temperature range. However, a pronounced thermal decomposition reaction commences at 419 °C, as evidenced by a significant increase in the rate of weight loss. This rate continues to rise with increasing temperature, reaching a maximum at 437 °C, after which it declines progressively until the decomposition process is complete. At a higher heating rate of 20 °C/min, the TG curve remains linear up to 295 °C, beyond which a distinct thermal decomposition reaction is observed. The rate of weight loss increases steadily from 438 °C, peaking at 479 °C, and subsequently diminishes until the reaction concludes. These findings highlight the influence of heating rate on the onset and progression of thermal decomposition in FVMQ, with higher heating rates accelerating the decomposition process and shifting the peak temperature to higher values.

**Figure 11:**
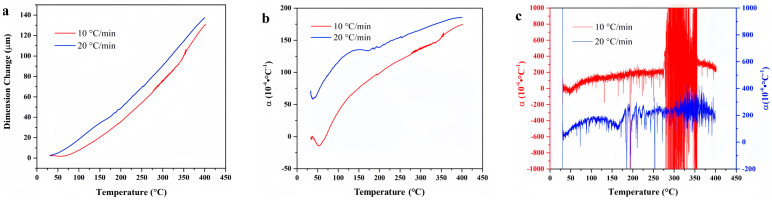
Temperature-deformation curve, temperature-average linear thermal expansion coefficient curve and temperature-differential linear thermal expansion coefficient curve of FVMQ under varying heating rates. (**a**) Temperature-deformation curves; (**b**) Temperature-average linear coefficient of thermal expansion curves; (**c**) Temperature-differential linear thermal expansion coefficient curves.

As illustrated in Figure 11, the TG curves obtained at varying heating rates exhibit similar profiles, while the corresponding DTG curves each display a single peak. This observation indicates that FVMQ undergoes a singular thermal decomposition mechanism, specifically the scission of the main chain. At a heating rate of 10 °C/min, the TG curve remains nearly linear up to 355 °C, indicating minimal weight loss and thermal stability within this temperature range. However, a pronounced thermal decomposition reaction commences at 419 °C, as evidenced by a significant increase in the rate of weight loss. This rate continues to rise with increasing temperature, reaching a maximum at 437 °C, after which it declines progressively until the decomposition process is complete. At a higher heating rate of 20 °C/min, the TG curve remains linear up to 295 °C, beyond which a distinct thermal decomposition reaction is observed. The rate of weight loss increases steadily from 438 °C, peaking at 479 °C, and subsequently diminishes until the reaction concludes. These findings highlight the influence of heating rate on the onset and progression of thermal decomposition in FVMQ, with higher heating rates accelerating the decomposition process and shifting the peak temperature to higher values.

### 4.5. NBR 5080

**Figure 12:**
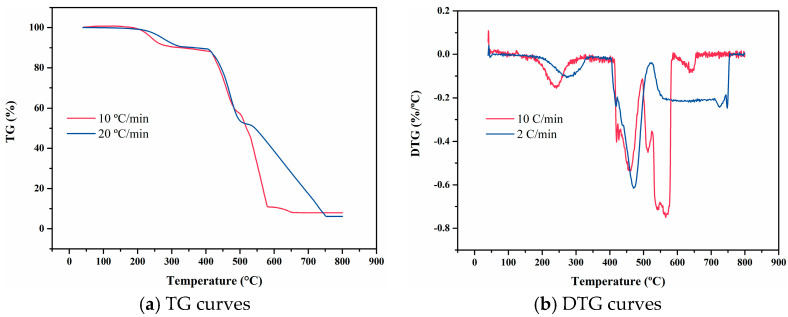
TG and DTG curves of NBR 5080 at varying heating rates.

As depicted in Figure 12, the weight loss trends of NBR exhibit similar profiles at heating rates of 10 °C/min and 20 °C/min. The TG curve of NBR 5080 demonstrates typical multi-step weight loss characteristics. Increasing the heating rate induces a hysteresis phenomenon, which results in a lower resolution of the TG curve. Specifically, the distinction between adjacent weight loss plateaus becomes indistinct, the peak of the DTG curve may be obscured, and the relevant pyrolysis characteristic parameters shift towards higher temperatures. At a heating rate of 10 °C/min, the weight loss reaction of NBR 5080 can be divided into three distinct stages. In the first stage (210–300 °C), the mass loss is approximately 10%, primarily attributed to the volatilization of additives. In the second stage (410–500 °C), the mass loss reaches 30%, mainly due to the scission of the macromolecular main chain and the random degradation of the material. In the third stage (510–590 °C), the mass loss is 45%, during which the material decomposes into acrylonitrile monomer and flammable gases.

**Figure 13:**
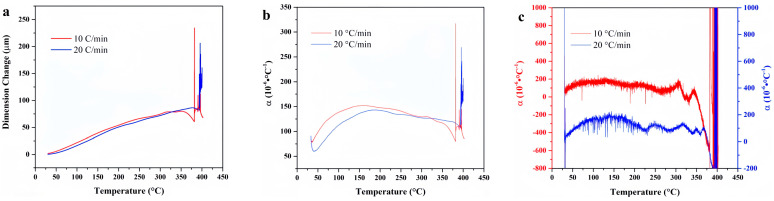
Temperature-deformation curves, temperature-average linear thermal expansion coefficient curves and temperature-differential linear thermal expansion coefficient curves of NBR 5080 under varying heating rates. (**a**) Temperature-deformation curves; (**b**) Temperature-average linear coefficient of thermal expansion curves; (**c**) Temperature-differential linear thermal expansion coefficient curves.

Figure 13 presents the temperature-deformation curve and the temperature-dependent linear thermal expansion coefficient curve of NBR 5080. The linear thermal expansion coefficient at a heating rate of 10 °C/min is higher than that at 20 °C/min. The thermal stability of NBR 5080 is significantly lower than that of the four fluororubbers previously discussed. This poor thermal stability is evidenced by the decline in the mean linear thermal expansion coefficient of NBR 5080 above 150 °C. When the temperature exceeds 350 °C, the material softens and continues to decompose, producing gas. This behavior further indicates that the thermal stability of NBR 5080 is markedly inferior to that of the other four fluororubbers.

### 4.6. SFB-1

**Figure 14:**
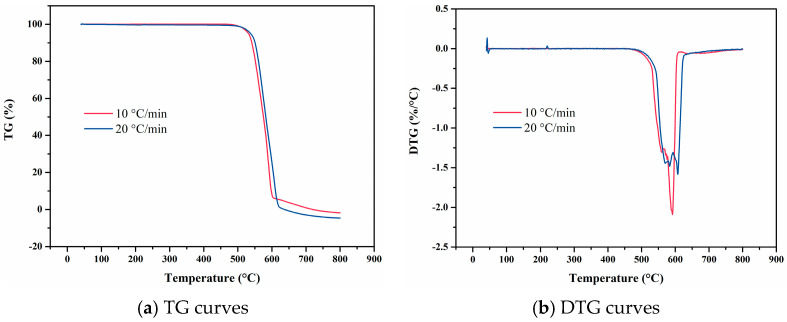
TG and DTG curves of polytetrafluoroethylene under varying heating rates.

As illustrated in Figure 14, SFB-1 exhibits high thermal stability, with minimal influence of heating rate on the weight loss temperature. When the heating rate is 10 °C/min, the TG curve remains nearly linear up to 502 °C. However, a pronounced thermal decomposition reaction commences at 540 °C. As the temperature increases, the rate of thermal decomposition weight loss gradually rises, reaching a peak at 591 °C, after which it declines progressively until the decomposition is complete. At a heating rate of 20 °C/min, the TG curve remains linear up to 498 °C. Beyond this temperature, a distinct thermal decomposition reaction is observed, starting at 551 °C. The rate of thermal decomposition weight loss increases steadily with temperature, peaking at 606 °C, and then diminishes until the reaction concludes. Notably, SFB-1 undergoes complete decomposition without residue when heated to 700 °C in air.

**Figure 15:**
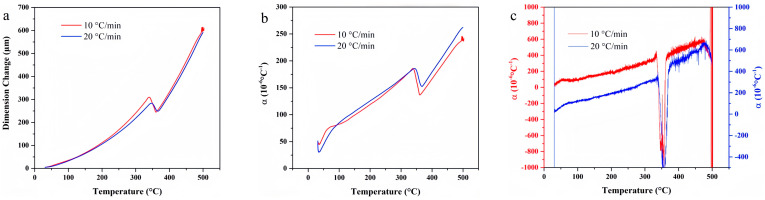
Temperature-deformation curves, temperature-average linear thermal expansion coefficient curves and temperature-differential linear thermal expansion coefficient curves of SFB-1 at varying heating rates. (**a**) Temperature-deformation curve; (**b**) Temperature-average linear coefficient of thermal expansion curve; (**c**) Temperature-differential linear thermal expansion coefficient curve.

Figure 15 presents the temperature-deformation curve and the temperature-dependent linear thermal expansion coefficient curve of SFB-1. In comparison with other elastomeric materials, the thermal expansion behavior of PTFE is characterized by distinct features. Specifically, a notable contraction is observed near 350 °C, which can be attributed to the softening and subsequent collapse of the material at elevated temperatures. Additionally, within the temperature range below 350 °C, PTFE exhibits a relatively low average linear thermal expansion coefficient, indicative of its superior dimensional stability under thermal stress.

### 4.7. SFB-2

**Figure 16:**
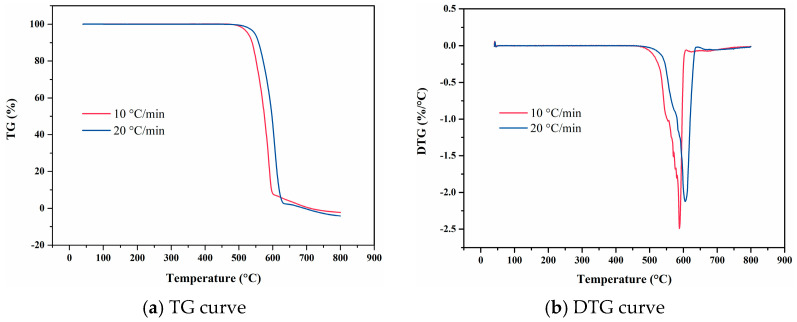
TG and DTG curves of SFB-2 under varying heating rates.

As illustrated in Figure 16, SFB-2 exhibits high thermal stability. The TG curves of SFB-2 and SFB-1 are nearly identical; however, the heating rate significantly influences the weight loss temperature of SFB-2. This discrepancy may be attributed to differences in processing methods and raw materials.

**Figure 17:**
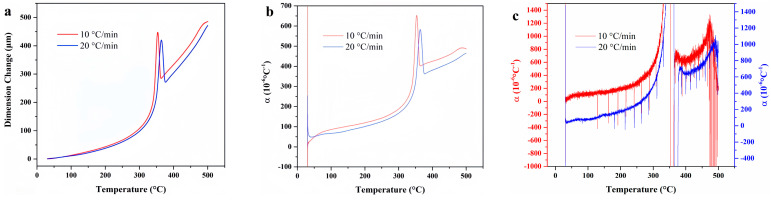
Temperature-deformation curves, temperature-average linear thermal expansion coefficient curves and temperature-differential linear thermal expansion coefficient curves of SFB-2 under varying heating rates. (**a**) Temperature-deformation curves; (**b**) Temperature-average linear coefficient of thermal expansion curves; (**c**) Temperature-differential linear thermal expansion coefficient curves.

Figure 17 presents the temperature-deformation curve and the temperature-dependent linear coefficient of thermal expansion of SFB-2. The thermal expansion behavior of SFB-2 closely resembles that of SFB-1. Upon heating above 300 °C, both materials exhibit rapid expansion, which can be attributed to the softening and subsequent collapse of the polymer structure. This phenomenon is indicative of the materials’ transition from a glassy to a rubbery state, characterized by a significant increase in molecular mobility and a corresponding loss of mechanical rigidity.

### 4.8. SZ7-3

**Figure 18:**
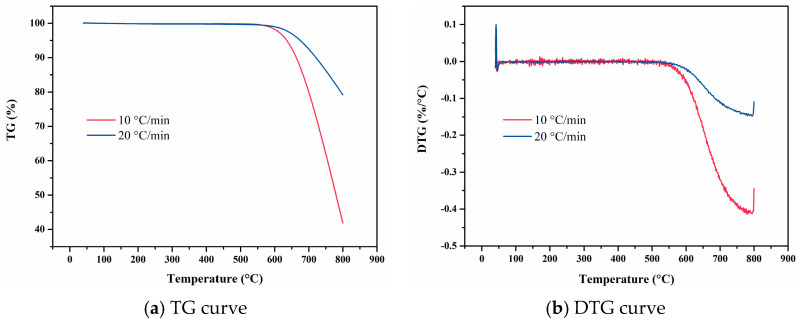
TG and DTG curves of ZC7-3 under varying heating rates.

As illustrated in Figure 18, the ZC7-3 exhibits high thermal stability, as evidenced by the similar profiles of the TG curves obtained at varying heating rates. However, increasing the heating rate shifts the TG-DTG curves towards higher temperature regions. At a heating rate of 10 °C/min, the TG curve remains nearly linear up to 582 °C, indicating minimal weight loss and thermal stability within this temperature range. Beyond 582 °C, the curve begins to deviate, and a more pronounced thermal decomposition reaction is observed starting at 635 °C. When the heating rate is increased to 20 °C/min, the TG curve remains nearly linear up to 599 °C, beyond which a more pronounced thermal decomposition reaction commences at 675 °C. These observations suggest that the thermal decomposition process is accelerated at higher heating rates, resulting in a shift of characteristic pyrolysis parameters towards higher temperatures.

**Figure 19:**
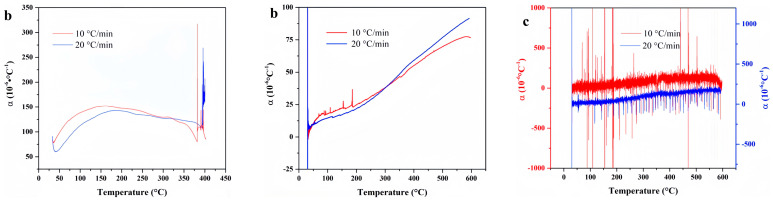
Temperature-deformation curves, temperature-average linear thermal expansion coefficient curves and temperature-differential linear thermal expansion coefficient curves of ZC7-3 under varying heating rates. (**a**) Temperature-deformation curve; (**b**) Temperature-average linear coefficient of thermal expansion curve; (**c**) Temperature-differential linear thermal expansion coefficient curve.

Figure 19 presents the temperature-deformation curve and the temperature-dependent linear thermal expansion coefficient curve of the graphite gasket. In comparison with other polymer materials, graphite exhibits a significantly lower linear thermal expansion coefficient, primarily attributed to its unique crystalline structure. This structural characteristic imparts exceptional thermal stability and dimensional integrity to graphite, even under elevated temperatures. Additionally, the absence of an obvious thermal hysteresis phenomenon further underscores the material’s consistent thermal behavior, which is highly desirable for applications requiring precise temperature control and minimal deformation.

### 4.9. Data Comparison

**Figure 20:**
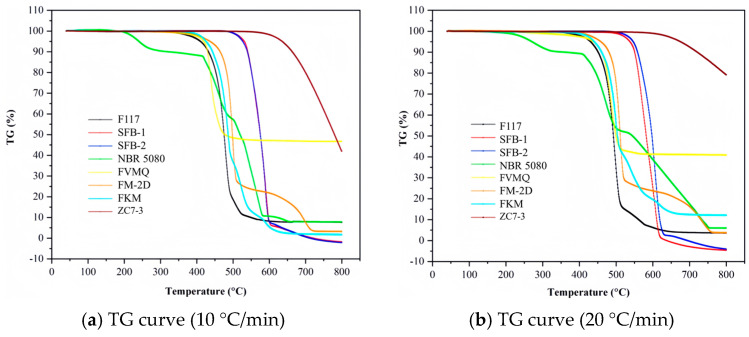
TG curves of non-metallic materials at varying heating rates.

Figure 20 presents the TG curves of various non-metallic materials at different heating rates, while Table 7 provides a detailed summary of the pyrolysis characteristics of these eight materials under varying heating conditions. Through a comprehensive comparison of these characteristics, the relative thermal stability of the materials can be ranked as follows: graphite exhibits the highest thermal stability, followed by SFB-1 and SFB-2. These materials are more thermally stable than FM-2D, FKM, F117, and FVMQ, which in turn are more stable than NBR 5080. This ranking highlights the significant differences in thermal stability among these materials, with graphite demonstrating the highest stability and NBR 5080 exhibiting the lowest.

The TGA curves of various materials at two different heating rates (10 °C/min and 20 °C/min), reflect the mass change behavior of these materials during the heating process. The horizontal axis represents temperature, ranging from 0 °C to 800 °C, while the vertical axis indicates the percentage of mass loss, ranging from −10% to 110%. Each curve represents the mass change of material during heating, with the percentage of mass loss reflecting the reduction in mass due to decomposition, oxidation, or volatilization caused by physical or chemical changes. At a heating rate of 10 °C/min, the thermal decomposition behavior of the materials is relatively mild, with lower onset temperatures and rates of mass loss. For example, F117 exhibits minimal mass loss at 800 °C, indicating high thermal stability, whereas ZC7-3 shows significant mass loss at lower temperatures, suggesting lower thermal stability. However, at a heating rate of 20 °C/min, the rapid temperature change within a unit of time means that the internal heat transfer and reaction rates within the materials may not keep pace with the external temperature increase. This results in higher onset temperatures for mass loss and changes in the curve profiles. For instance, FKM may undergo significant decomposition between 300 °C and 400 °C at 10 °C/min, but this process may be delayed to between 400 °C and 500 °C at 20 °C/min. The variation in heating rate significantly affects the thermal behavior of materials, with rapid heating potentially causing decomposition or phase transitions at higher temperatures. Data in Table 7 further quantify the decomposition characteristics of these materials. For example, F117 has an onset temperature (*T*1%) of 346 °C for 1% mass loss at a heating rate of 10 °C/min, while NBR 5080 has a *T*1% of only 207 °C, indicating that F117 is more stable at high temperatures. Additionally, SFB-1 and SFB-2 exhibit higher decomposition temperatures at elevated temperatures, suggesting better performance under extreme conditions.

**Figure 21:**
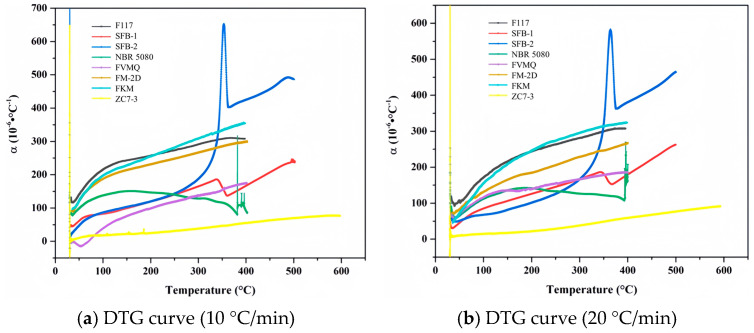
Curves of temperature-average linear thermal expansion coefficient of non-metallic materials at varying heating rates.

Figure 21 presents the curves of the average linear thermal expansion coefficient versus temperature for various non-metallic materials at different heating rates, while Table 8 lists the corresponding average linear thermal expansion coefficients for these materials. Combining these data, we can conduct a comprehensive analysis of the thermal expansion behavior of these materials under varying heating rates (10 °C/min and 20 °C/min). The figures illustrate how the linear thermal expansion coefficients (α) of these materials vary with temperature during heating, while the tables provide specific values of α within the temperature range of 100 °C to 350 °C (expressed in units of 10^−6^ °C^−1^). Generally, materials with lower thermal expansion coefficients prior to thermal decomposition exhibit smaller thermal deformations and superior dimensional stability. For example, graphite and PTFE show minimal decomposition below 400 °C and have very low thermal expansion coefficients, indicating excellent dimensional stability. In contrast, other fluoropolymers such as F117 and FKM exhibit higher expansion coefficients and partial decomposition between 300 °C and 400 °C. Therefore, the comparison of average linear thermal expansion coefficients is limited to the temperature range from room temperature to 300 °C. Integrating the data from Figure 21 and Table 8, the eight non-metallic materials can be categorized based on their average linear thermal expansion coefficients. Graphite demonstrates the lowest coefficient, followed by FVMQ and two types of PTFE (SFB-1 and SFB-2), which exhibit moderate coefficients. NBR 5080 has a higher coefficient than the aforementioned materials but is more stable than FM-2D, FKM, and F117. The heating rate significantly influences the thermal expansion behavior. At 20 °C/min, the expansion coefficients are generally lower than those at 10 °C/min, suggesting that rapid heating may suppress the expansion behavior of materials. However, certain materials exhibit unique behaviors under rapid heating conditions. For instance, the expansion coefficient of FM-2D at 20 °C/min is significantly reduced, while SFB-2 shows a marked increase in its expansion coefficient at high temperatures (e.g., α drops from 584 to 319 at 350 °C), indicating possible phase transitions or structural changes. Additionally, FVMQ has a relatively low expansion coefficient at 10 °C/min but experiences a significant increase at 20 °C/min, highlighting the pronounced effect of heating rate on its thermal expansion behavior. NBR 5080 exhibits a decreasing expansion coefficient with increasing temperature at 10 °C/min, while its coefficient remains relatively stable at 20 °C/min, suggesting that its thermal expansion behavior stabilizes under rapid heating conditions. In summary, the thermal expansion behavior of materials is influenced not only by their intrinsic properties but also by the heating rate. In practical applications, material selection should consider the linear thermal expansion coefficient, stability under different heating rates, and dimensional stability at high temperatures to ensure reliability and performance in specific operating conditions.

## 5. Material Fire Test Verification

**Figure 22:**
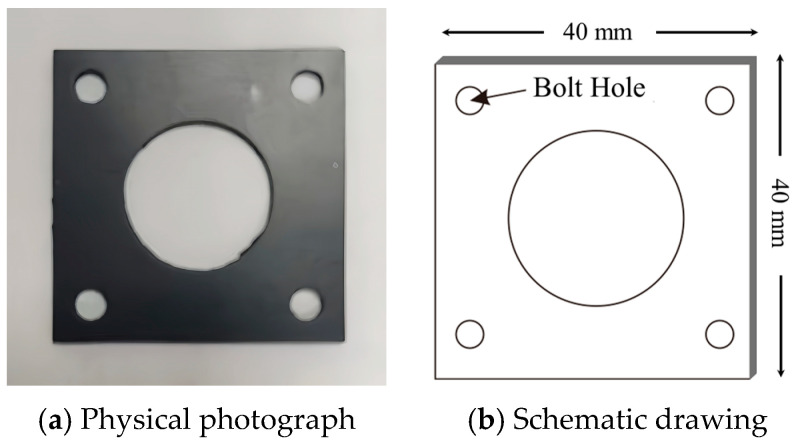
Fireproof test object.

To directly verify the thermal physical performance test results of non-metallic fireproof sealing materials, this study selected a typical material from each of the four types of non-metallic materials and conducted fireproof tests on non-metallic pipe connections under conditions simulating oil flow. This approach aimed to assess the fireproof sealing performance of different non-metallic materials in fuel and lubricating oil lines. The dimensions of the test materials were standardized at 2 mm × 40 mm × 40 mm, as illustrated in Figure 22.

**Figure 23:**
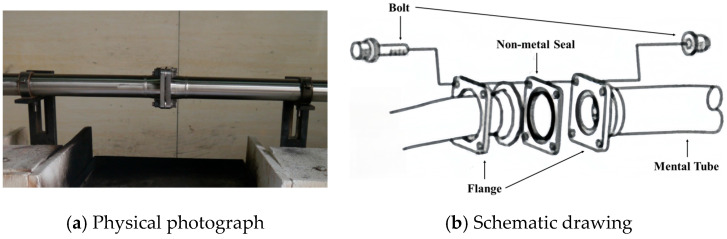
Schematic diagram of non-metallic material fireproof test tooling and test parts.

The metal pipe, featuring a face-sealed flange joint, is constructed from 321 stainless steel. The pipe has a length of 600 mm, an outer diameter of 19 ± 1 mm, and a wall thickness of 0.9 ± 0.1 mm. The tightening torque for the flange joint is specified as 10.1 ± 1 Nm. The testing setup involving non-metallic materials and stainless-steel metal pipes is depicted in Figure 23.

In the aero-engine oil circuit system, the fuel operates at pressures ranging from 2 to 13 MPa, with a maximum pressure of approximately 5 MPa under idle conditions. The working temperature is maintained below 160 °C, and the reference flow rate of the main fuel supply pipeline is 2.3 L/min. The lubricating oil system operates at pressures between 0.3 and 0.7 MPa, with a maximum limit of 2.1 MPa. Its working temperature ranges from 60 °C to 160 °C, and transient temperatures do not exceed 175 °C. Under idle conditions, the distribution flow rate of the circulating pipeline ranges from 0.65 to 10 L/min, with windmilling condition flow rates calculated proportionally based on high-pressure rotor speeds. To evaluate the fireproof of non-metallic pipe connections, four sets of tests were conducted, each including pipelines for aviation lubricating oil and aviation kerosene. The test conditions were selected based on actual operating conditions and fireproof test data, optimizing a typical set of inlet temperature, pressure, and flow rate within the specified ranges. For aviation kerosene, the verification parameters included an inlet temperature of 120 °C, a medium pressure of 5 MPa, and an inlet flow rate of 130 L/h. For aviation lubricating oil, the parameters were an inlet temperature of 120 °C, a medium pressure of 0.5 MPa, and an inlet flow rate of 60 L/h, as detailed in Table 9. Before testing, the tightening torque of the test piece was determined. The test piece was then exposed to a standard flame for either 5 or 15 min, during which the fluid temperature, pressure, and flow rate through the test piece were measured in real time. Temperature sensors, pressure sensors, and mass flowmeters were installed at both the inlet and outlet of the test piece to monitor changes in fluid parameters. During the test, the fluid parameters were controlled within the following accuracies: temperature ±2 °C, flow rate ±5 L/h, and pressure ±100 kPa.

**Figure 24:**
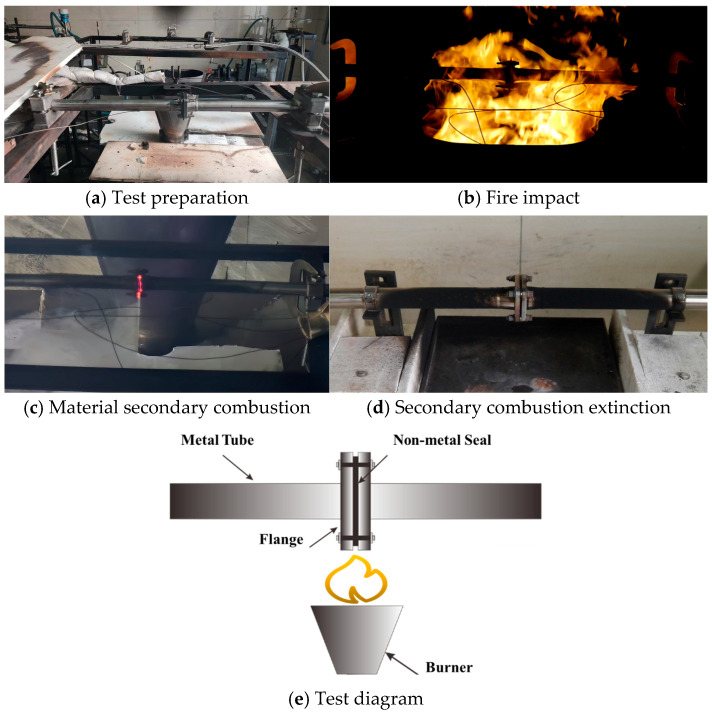
Fireproof test process diagram.

Figure 24 illustrates the entire testing process, encompassing test preparation, exposure to fire, secondary combustion of the material, and the subsequent extinguishment of this secondary combustion.

**Figure 25:**
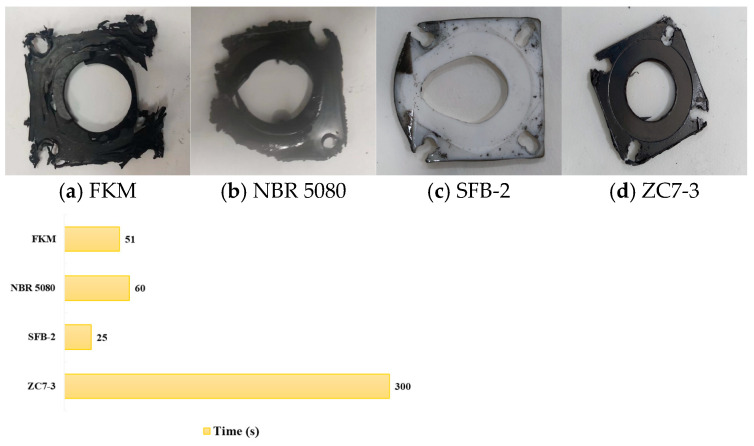
Pipeline leakage time of RP-3 jet fuel as the flowing medium.

When the medium in the tube is RP-3, FKM leaks oil in approximately 51 s. After the test, the edge of the fluorocarbon rubber test specimen is severely damaged, with an obvious fracture at the opening position. NBR 5080 leaks in about 60 s. After the test, the nitrile rubber specimen shows significant heat loss, carbonization, residual embrittlement, and severe deformation of the middle channel structure. SFB-2 leaks oil in about 25 s. After the test, the polytetrafluoroethylene specimen is softened at high temperature, with complete deformation of the fixed holes and the middle oil channel. Graphite does not leak oil within 300 s, and the graphite gasket shows no significant changes after the test. The pipeline leakage time is illustrated in Figure 25.

**Figure 26:**
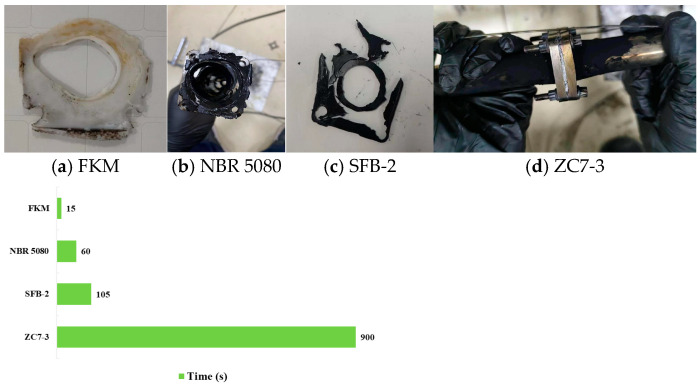
Pipeline leakage time of Mobil Jet™ Oil II as the flowing medium.

In the case of Mobil Jet™ Oil II, FKM leaked oil in approximately 15 s. Post-test observations revealed that the fluorocarbon rubber specimen was completely damaged; however, the size of the middle hole remained largely unchanged, and the residual portion retained some degree of toughness. NBR 5080 leaked in about 60 s. After the test, the NBR sample exhibited significant heat loss and carbonization, resulting in low residual strength and complete loss of toughness. SFB-2 leaked oil after approximately 105 s. Post-test, the polytetrafluoroethylene specimen showed severe blistering at the edges, and the fixed holes and middle oil channel were completely deformed due to high-temperature collapse. The SZ7-3 did not leak oil for 900 s and showed no significant changes after the test. The pipeline leakage times are illustrated in Figure 26.

## 6. Conclusions

This study comprehensively investigated the thermal and mechanical properties of non-metallic materials utilized in critical components of aero engines, with a focus on their suitability for high-temperature and fire-resistant applications. The key findings are summarized as follows:

(1) Experimental results revealed that ZC7-3 demonstrated the highest thermal decomposition temperature among the tested materials, underscoring its exceptional thermal stability. Polytetrafluoroethylene variants SFB-1 and SFB-2 also exhibited robust high-temperature resistance, with decomposition temperatures exceeding 500 °C. Fluorine-based elastomers, including F117, FKM, and FM-2D, displayed relatively high decomposition temperatures, indicating their potential for stability in elevated-temperature environments. In contrast, NBR 5080 exhibited the lowest thermal decomposition temperature, highlighting its limited thermal stability under extreme conditions.
(2) ZC7-3 exhibited the lowest linear thermal expansion coefficient, establishing it as the material with the most superior dimensional stability. Polytetrafluoroethylene and FVMQ also demonstrated favorable thermal expansion properties, maintaining dimensional integrity even at elevated temperatures. While F117, FKM, and FM-2D exhibited higher thermal expansion coefficients, they remained dimensionally stable within a defined temperature range. Conversely, NBR 5080 displayed significant dimensional changes at high temperatures, rendering it unsuitable for applications requiring precise dimensional control.
(3) Based on the combined assessment of thermal decomposition temperature and dimensional stability, ZC7-3, polytetrafluoroethylene, and FVMQ emerged as the most promising candidates for aero engine sealing applications. These materials not only exhibited exceptional high-temperature resistance but also maintained remarkable dimensional stability, thereby enhancing the operational safety and reliability of aero engines.
(4) Fireproof performance tests were conducted on four representative non-metallic materials, referencing their thermal physical properties. The results indicated that ZC7-3 exhibited unparalleled fireproof performance, maintaining both dimensional stability and sealing integrity during 5-min and 15-min fire exposure tests. In contrast, FKM, NBR 5080, and polytetrafluoroethylene were deemed unsuitable for the static seal configuration employed in this study, as they failed to meet the required performance criteria under fire conditions.
(5) To further validate the suitability of these materials for aero-engine applications, future studies should investigate their long-term performance under cyclic thermal and mechanical loading conditions. Additionally, the development of advanced composite materials, integrating the thermal stability of ZC7-3 with the flexibility of fluoropolymers, could offer a promising avenue for enhancing both performance and durability in extreme environments.

## Figures and Tables

**Table 1:** Main performance parameters of various types of rubber.

No.	Type	Name	Tensile Strength (MPa)	Elongation at Break (%)	Compression Set (%)	Shore A (HA)	Standard
1	Fluororubber	F117	11	191	(200 °C/24 h/25%) ≤ 22	64	Q/6S1822-2002 [26]
2	Fluorocarbon rubber	FKM	≥15	≥120	(200 °C/24 h/25%) ≤ 35	70~80	SAE AMS 7287 [27]
3	Fluoroelastomers	FM-2D	9	110	(250 °C/24 h/20%) ≤ 50	69	Q/6S 1590-2010 [28]
4	Fluorosilicone rubber	FVMQ	≥5.17	≥125	(175 °C/22 h/25%) ≤ 35	50~80	SAE AMS-R-25988 [29]
5	Nitrile butadiene rubber	NBR 5080	10~25	200~600	(100 °C/24 h/25%) ≤ 40	50~90	GJB5037-2001 [30]

**Table 2:** Main performance parameters of polytetrafluoroethylene.

No.	Type	Name	Tensile Strength (MPa)	Elongation at Break (%)	Compression Set (%)	Shore A (HA)	Standard
1	Polytetrafluoroethylene	SFB-1	20~35	200~400	5~15	50~65	ASTM D4894/GB/T 20671 [31,32]
2	Polytetrafluoroethylene	SFB-2	25~40	250~500	5~10	55~70	ASTM D4894/GB/T 20671

**Table 3:** Main properties of ZC7-3.

Graphite	Volume Density (g/cm^3^)	Shore’s Hardness (Hs)	Break Resistant Strength (MPa)	Compression Resistance Strength (MPa)	Porosity (%)	Impact Resistance Strength (10^−5^ J/mm)	Elasticity Modulus (GPa)	Highest Make with Temperature (°C)
ZC7-3	1.90	100	100	250	0.5	4	15	500

**Table 4:** Technical parameters of TGA [33].

Parameter	Value
Temperature Data	
Temperature Range	Room temperature to 1100 °C
Temperature Accuracy	±1 °C
Temperature Precision	±0.6 °C
Heating Rate	0.02~150 °C/min
Cooling Time	20 min (1100~100 °C)
Helium Cooling Time	≤11 min (1100~100 °C)
Balance Data	
Measurement Range	≤5 g
Resolution	0.1 μg
Weighing Accuracy	0.005%
Weighing Precision	0.0025%
Repeatability	<0.0009 mg
Typical Minimum Weighing Value	0.17 mg
Blank Curve Reproducibility	>±10 μg

**Table 5:** Technical parameters of the TMA [34].

Parameter	Value
Temperature Range	−150~1000 °C
Temperature Precision	±1 °C
Cooling Time	From 600 °C to 50 °C <10 min
Measurement Precision	±0.1%
Sensitivity	15 nm
Displacement Resolution	<0.5 nm
Dynamic Baseline Drift	<1 μm (100~500 °C)
Force Range	0.001~2 N
Force Resolution	0.001 N
Frequency Range	0.01~2 Hz

**Table 6:** Calibration data for temperature and heat flux of the Carlin 201 CRD burner flame.

No.	1	2	3	4	5	6	7
*T* (°C)	1045	1073	1086	1065	1049	1022	1025
*T*_ave_ (°C)		1052		*q*_ave_ (W/cm^2^)		11.0	

**Table 7:** Pyrolysis characteristics of non-metallic materials at varying heating rates.

Sample	(°C/min)	*T*1% (°C)	*T*5% (°C)	*T*10% (°C)	*T*50% (°C)	*Te* (°C)	*Tm* (°C)
F117	10	346	410	432	475	448	477
	20	371	430	451	493	476	498
FKM	10	391	425	443	486	449	485
	20	395	441	460	504	474	500
FM-2D	10	376	436	464	498	483	499
	20	404	458	481	511	493	512
FVMQ	10	355	419	430	477	425	437
	20	295	438	460	500	457	479
NBR 5080	10	207	241	325	519	——	241
	20	209	271	367	544	——	273
SFB-1	10	502	531	540	575	539	591
	20	498	538	551	583	554	606
SFB-2	10	500	526	539	576	553	588
	20	514	547	559	598	576	607
ZC7-3	10	582	635	663	779	——	——
	20	599	675	721	——	——	——

**Table 8:** Average linear thermal expansion coefficients of non-metallic materials at varying heating rates.

Sample	(°C/min)	α100 (10^−6^ °C^−1^)	α150 (10^−6^ °C^−1^)	α200 (10^−6^ °C^−1^)	α250 (10^−6^ °C^−1^)	α300 (10^−6^ °C^−1^)	α350 (10^−6^ °C^−1^)
F117	10	215	243	257	273	291	307
	20	171	216	243	263	280	299
FKM	10	197	227	254	280	308	333
	20	154	208	244	272	294	313
FM-2D	10	188	214	231	249	267	286
	20	131	166	184	206	229	247
FVMQ	10	39	76	99	119	136	154
	20	116	135	139	155	166	178
NBR 5080	10	139	152	147	140	129	117
	20	112	136	140	132	127	123
SFB-1	10	82	99	119	140	165	163
	20	85	106	125	145	167	182
SFB-2	10	86	103	119	143	188	584
	20	67	82	101	125	167	319
ZC7-3	10	18	22	25	31	38	47
	20	15	17	22	29	38	49

**Table 9:** Fireproof test conditions.

Serial Number	Fluid Medium	Material Grade	Temperature (°C)	Pressure (MPa)	Flow Rate (L/h)	Fire Impact Time (min)
1	RP-3	FKM	120	5	130	5
2		NBR 5080				
3		SFB-2				
4		ZC7-3				
5	Mobil Jet™ Oil II	FKM	120	0.5	60	15
6		NBR 5080				
7		SFB-2				
8		ZC7-3				

## Data Availability

The original contributions presented in the study are included in the article, further inquiries can be directed to the corresponding author.
